# Evaluation of the Prevalence of *Staphylococcus aureus* in Chicken Fillets and Its Bio-Control Using Different Seaweed Extracts

**DOI:** 10.3390/foods12010020

**Published:** 2022-12-21

**Authors:** Gamal Hamad, Amr Amer, Ghada Kirrella, Taha Mehany, Reham A. Elfayoumy, Rasha Elsabagh, Eman M. Elghazaly, Tuba Esatbeyoglu, Ahmed Taha, Ahmed Zeitoun

**Affiliations:** 1 Department of Food Technology, Arid Lands Cultivation Research Institute, City of Scientific Research and Technological Applications, New Borg El-Arab 21934, Egypt; 2 Department of Food Hygiene and Control, Faculty of Veterinary Medicine, Alexandria University, Alexandria 21544, Egypt; 3 Department of Food Control, Faculty of Veterinary Medicine, Kafrelsheikh University, Kafrelsheikh 33516, Egypt; 4 Department of Botany and Microbiology, Faculty of Science, Damietta University, Damietta 34511, Egypt; 5 Department of Food Hygiene and Control, Faculty of Veterinary Medicine, Benha University, Qaluobia 13736, Egypt; 6 Department of Microbiology, Faculty of Veterinary Medicine, Matrouh University, Matrouh 51511, Egypt; 7 Department of Food Development and Food Quality, Institute of Food Science and Human Nutrition, Gottfried Wilhelm Leibniz University Hannover, Am Kleinen Felde 30, 30167 Hannover, Germany; 8 Department of Food Science, Faculty of Agriculture (Saba Basha), Alexandria University, Alexandria 21531, Egypt; 9 Department of Functional Materials and Electronics, Center for Physical Sciences and Technology, Saulėtekio al. 3, 10257 Vilnius, Lithuania

**Keywords:** chicken safety, natural antimicrobials, natural antioxidants, natural preservatives, phenolics, *S. aureus*, seaweed extracts, shelf-life

## Abstract

This study aims to assess the occurrence of *Staphylococcus aureus* in chicken fillets and to control its growth using various lyophilized seaweed extracts (i.e., *Halimeda opuntia* (HO), *Actinotrichia fragilis*, and *Turbinaria turbinata*) by an agar disk diffusion assay in vitro. Results showed that prevalence of *S. aureus* in breast and thigh samples reached of 92% and 84%, respectively. Lyophilized HO extract was the only seaweed that showed the antibacterial activity against *S aureus* with a significant difference at *p* < 0.05. The minimum inhibitory concentration (MIC) of HO extract was 1.5%, with an inhibition zone of 8.16 ± 0.73 mm. Regarding 1,1-diphenyl-2-picrylhydrazyl (DPPH) scavenging activity, IC_50_ was recorded at 55.36 μg/mL, whereas cytotoxic IC_50_ of the lyophilized HO extract on peripheral blood mononuclear cells (PBMCs) was 33.7 µg/mL; a higher IC_50_ of HO extracts permits their use as a safe food additive in meat products. Moreover, total phenolic compounds and total flavonoids compounds recorded 20.36 ± 0.092 and 16.59 ± 0.029 mg/mL, respectively. HPLC analyses of phenolic compounds profiles exhibited many bioactive substances and the higher ratio was daidzein with 10.84 ± 0.005 µg/mL and followed by gallic acid with a value of 4.06 ± 0.006 µg/mL. In a challenge study, chicken fillet (CHF) experimentally inoculated with *S. aureus* (ST) and treated with the lyophilized HO algal extract at 4% and 6% (CHF/ST/HO) showed a complete reduction of *S. aureus* count on the 6th and 4th days in chicken fillet stored at 4 °C, respectively. Moreover, CHF/ST/HO at 4% and 6% of HO extract enhanced the sensory attributes of grilled un-inoculated chicken fillet. Thus, lyophilized HO extracts are promising antibacterial and antioxidant candidates in the chicken meat industry.

## 1. Introduction

Food safety is a top priority for both public health and the economy. Approximately 1 out of 10 yearly suffers from food poisoning from contaminated food consumption [1]. Providing high-quality, safe, and nutritious food will become increasingly difficult in the next decades [2], as both nutrition and food safety are interlinked for health outcomes from food systems [3].

Chicken meat products are commonly recognized as an important source of protein worldwide. In 2018, world poultry production reached 123 million tons in 1 year, with a prediction to increase [4]. However, chicken meat products commonly harbor *S. aureus*, a food poisoning bacterium [5] and a pathogen that contaminates food during handling and processing [6]. It is a Gram-positive, facultative anaerobe, and toxic bacterium [7]. *S. aureus* is a major concern in chicken meat and shows resistance to various antibiotic types, even methicillin [8]. Recent research has focused on using natural antimicrobials in meat products [9]. A novel natural antimicrobial that has antibacterial impacts against *S. aureus* is seaweed (macroalgae). Marine algae are consumed as food worldwide and are used to extract polysaccharides and gelatinous substances [10]. Moreover, it is considered a functional diet rich in antioxidants, phenolic acids, flavonoids, pigments, protein, vitamins, essential amino acids, minerals, fats, polysaccharides, and proteins. Hence, the antioxidative characteristics of several algae have been analyzed in recent studies by different in vitro and in vivo assays [11,12]. Moreover, algae enhance the antioxidant properties of chicken meat [13]. However, there is a need for further information and the application of seaweeds as food [14], in particular their cytotoxity.

This study aimed to assess the following topics: (I) prevalence of *S. aureus* infection in the breast and thigh of chicken meat samples; (II) in vitro anti-*S. aureus* effect of three lyophilized seaweed extracts (i.e., *Halimeda opuntia*, *Actinotrichia fragilis*, and *Turbinaria turbinata*); (III) MIC, DPPH scavenging activity, cytotoxicity, TFC, TPC, HPLC phenolic profile of lyophilized HO extract; and (IV) antibacterial capability of lyophilized HO extract in chicken fillet experimentally inoculated with *S. aureus* concerning sensory attributes of grilled un-inoculated chicken fillet.

## 2. Materials and Methods

### 2.1. Collection of Chicken Fillet and Determination of S. aureus

A total of 100 chicken breast and thigh samples were collected from several local markets in Alexandria Governorate, Egypt. The chicken meat was collected randomly from the local retails which sold in pieces, at refrigerated temperature, and packaged in polyethylene bags. These samples were transferred to the laboratory in an ice box to be bacteriologically examined without delay. Isolation of *S. aureus* was applied in Baird Parker selective media in duplicate at 37 °C after 24 h [15].

### 2.2. Bacterial Strain

Pathogenic *S. aureus* EMCC 1351 was obtained from Microbiological Resources Center (MERCIN), Faculty of Agriculture, Ain Shams University, Cairo, Egypt. Bacterial strain was prepared and adjusted at a bacterial density of 1 × 10^7^ CFU/mL according to Eldin et al. [16].

### 2.3. Algal Materials and Extraction

**Figure 1:**
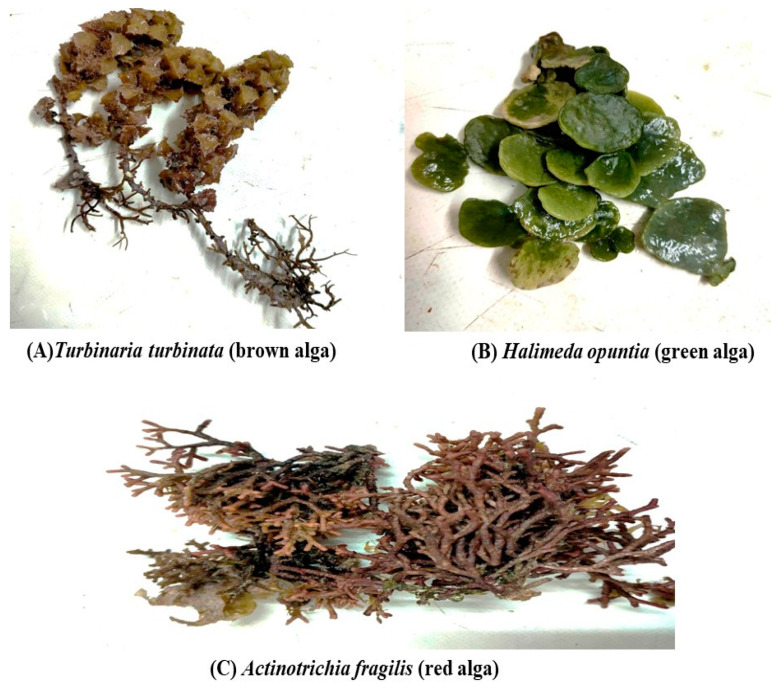
Pictures of the seaweeds investigated in the study: (**A**) *Turbinaria turbinata* (TT) (brown alga); (**B**) *Halimeda opuntia* (HO) (green alga), and (**C**) *Actinotrichia fragilis* (AF) (red alga).

Three seaweeds were collected from Hurghada city, Red Sea Governorate, Egypt i.e., *Halimeda opuntia* (HO) (green algae), *Actinotrichia fragilis* (AF) (red algae), and *Turbinaria turbinata* (TT) (brown algae) (see Figure 1). Algal species were carefully cleaned from the epiphytes and then dried and powdered. Each algal powder was prepared as a lyophilized ethanolic extract (70% ethanol: deionized water *v*/*v*). The identification of the seaweed species was carried out according to Salem et al. [17] and Yang et al. [18].

### 2.4. Antibacterial Activity

#### 2.4.1. Assessment of the Antibacterial Activity of Lyophilized Seaweed Extracts

The ability of lyophilized seaweed extracts as an antibacterial against *S. aureus* reference strain EMCC1351 (prepared in Microbiological Resources Center (MERCIN), Faculty of Agriculture, Ain Shams University, Cairo, Egypt) was evaluated using agar disk diffusion assay [19,20]. Overnight culture of *S. aureus* was enriched on Mueller Hinton Medium (MHM) broth (Oxoid, Cheshire, UK) at 37 °C/48 h and then spread on MHM plates. After dryness, the lyophilized seaweed extracts were loaded onto each separate disk (20 µL), and the plates were maintained at 4 °C/30 min and then incubated at 37 °C/24 h. The clear inhibitory zones obtained were recorded in mm, considering the anti-*S. aureus* activity of various lyophilized seaweed extracts. In addition, the results of the inhibitory zones were compared with those of three antibiotic disks, tetracycline, chloramphenicol, and sulfamethoxazole.

#### 2.4.2. Evaluation of the Minimum Inhibitory Concentrations (MICs) of Lyophilized HO Extract

On the basis of the antibacterial activity results of the three seaweed extracts, we evaluated MIC for the only one that has antibacterial power against *S. aureus*, thus, HO algal extracts minimum inhibitory concentrations against *S. aureus* were evaluated according to Kadaikunnan et al. [21] using descending concentrations. It was performed using different concentrations, i.e., 100, 50, 25, 12.5, 6.25, 3.12, 1.56, and 0.78 mg/mL lyophilized HO algal extract. *S. aureus* suspension of grown cultures was prepared and adjusted to a density of 10^6^ colony forming unit (CFU)/mL [16].

### 2.5. Phytochemical Analysis of the Lyophilized HO Algal Extract

#### 2.5.1. Assessment of the Radical Scavenging Capacity by the DPPH Assay

The ability of the lyophilized HO algal extract to scavenge DPPH free radicals was assessed according to Catarino et al. [22] and Hamad et al. [23] with few modifications. Ascorbic acid was used as a positive control. Values were expressed as IC_50_ (the lyophilized HO extract’s concentration inhibited 50% DPPH). IC_50_ values were compared with a concentration plot using a nonlinear regression algorithm. Inhibition % was calculated according to Equation (1).

(1) $$\mathrm{Inhibition}\;\left(\%\right)=\frac{A\;\mathrm{of}\;\mathrm{control}\;-\;A\;\mathrm{of}\;\mathrm{the}\;\mathrm{sample}}{A\;\mathrm{of}\;\mathrm{control}}\;\times 100\;$$

where: A = absorbance.

#### 2.5.2. Total Phenolic and Total Flavonoid Contents of Lyophilized HO Algal Extract

Total phenolic content (TPC) of lyophilized HO algal extract (green algae) was evaluated by Folin-Ciocalteu technique at 765 nm using a UV/Vis spectrophotometer (PG Instrument Ltd. Lutterworth, UK) as follow; 1 mL of an 70% ethanolic HO extract (1%) was added to 0.1 mL of Folin-Ciocalteu reagent. The mixture was left for 15 min at room temperature. Then, 3 mL of 2% Na_2_CO_3_ was added. The prepared mixture was left for 30 min at room temperature before the measurement. The TPC was calculated using standard calibration curve of gallic acid, and TPC results was expressed in mg of gallic acid equivalents (GAE) per mL of HO extract (mg GAE/mL) [24]; hence, ethanol was used as blank sample.

On the other hand, the total flavonoid content (TFC) of lyophilized HO algal extract (green algae) were analyzed by a UV/Vis spectrophotometer at 510 nm. One milliliter of an 70% ethanolic HO extract (1%) was added to 4 mL of distilled H_2_O and mixed vigorously. After approximately 5 min, 0.3 mL of NaNO_2_ (5%) was added to the mixture and 0.3 mL of AlCl_3_ (10%) was added. Further, 2 mL of NaOH (1 M) after an extra 6 min was added and the volume of the prepared mixture was increased up to 10 mL of distilled H_2_O. TFC were expressed as mg of quercetin equivalent (QE) per mL of the HO extract (mg QE/mL) [25].

### 2.6. HPLC Evaluation of Phenolic Compounds Profiles of the Lyophilized HO Algal Extract

HPLC (Agilent 1260 infinity HPLC Series, Santa Clara, CA, USA) was used to screen lyophilized HO algal extract’s phenolic profile. Phenolic compounds were separated using an Eclipse C18 column (4.6 mm × 250 mm i.d., 5 μm) at 40 °C. The separation was achieved using a ternary linear elution gradient with (A): HPLC-grade water- 0.2% H_3_PO_4_ (*v*/*v*), obtained from Sigma-Aldrich, St. Louis, MO, USA; (B) methanol (Thermo Fisher Scientific, Waltham, MA, USA); and (C) acetonitrile (Thermo Fisher Scientific, Waltham, MA, USA). The mobile phase was applied at a 0.9 mL/min flow rate, and the multi-wavelength detector was adjusted to 280 nm. Approximately 5 μL injection volume was used. HPLC phenolic profile screening was conducted according to Hamad et al. [26].

### 2.7. Safety and Cytotoxicity Assay of Lyophilized HO Algal Extract

Lyophilized HO algal extract was evaluated for its effect on the viability of peripheral blood mononuclear cells (PBMCs). Cell viability was investigated using PBMCs maintained in Roswell Park Memorial Institute RPMI medium. To isolate PBMCs, whole blood, firstly diluted with PBS, was then gently layered over an equal volume of Ficoll in a Falcon and finally centrifuged for 30 min at 500 rpm without brake. Blank wells (150 µL PBS), control wells (150 µL PBMCs), and tested wells (150 µL PBMCs) were allocated on a 96-well microtiter plate. Lyophilized HO algal extracts at different concentrations were inoculated to test wells and then incubated for 24 h, according to Popiołkiewicz et al. [27]. Neutral red (150 µL) was added and then incubated at 37 °C for 2 h. After washing the cells, the plates were cleaned with a de-staining solution (1% acetic acid: 49% deionized water: 50% ethanol, 150 µL/well. At 540 nm, absorbance was monitored using a T80 UV/VIS spectrophotometer [28]. Lyophilized HO algal extract inhibition% was calculated using Equation (2) and IC_50_ values were calculated online: www.aatbio.com/tools/IC50-calculator, (accessed on 27 July 2022).

(2) $$\mathrm{Lyophilized}\;\mathrm{HO}\;\mathrm{algal}\;\mathrm{extract}\;\mathrm{inhibition}\%=100-\frac{O.D\;\mathrm{Control}\;-\;O.D\;\mathrm{Treatment}}{O.D\;\mathrm{Control}}$$

where O.D. = optical density; control = 150 µL PBMCs, treatment = 150 µL HO extract.

### 2.8. Assessment of the Antibacterial Effect of Lyophilized HO Algal Extract against S. aureus Experimentally Inoculated into Chicken Fillet

Raw chicken breast fillets (boneless) were sliced into 5 cm × 5 cm pieces using a sterile knife. Before the experiment, chicken fillets were sterilized with ultraviolet light (UV) for 15 min/side to control background micro-flora, according to Morsy et al. [29]. Prepared chicken fillet samples were divided into six groups as follows: Group 1, chicken fillet without any treatments (CHF); Group 2, chicken fillet treated with direct addition of lyophilized HO algal extract 4% (CHF/HO 4%); Group 3, chicken fillet treated with lyophilized HO algal extract 6% (CHF/HO 6%); Group 4, chicken fillet experimentally inoculated with 10^7^ CFU/mL *S. aureus* (CHF/ST); Group 5, chicken fillet experimentally inoculated with *S. aureus* and treated with lyophilized HO algal extract 4% (CHF/ST/HO 4%); and Group 6, chicken fillet experimentally inoculated with *S. aureus* and treated with lyophilized HO algal extract 6% (CHF/ST/HO 6%).

Samples were maintained at room temperature for 15 min to allow cell attachment after inoculation and were then chilled at 4 °C and examined bacteriologically every 2 days for *S. aureus* till the cells completely loss their viability. This experiment was repeated in triplicate to obtain the mean values for statistical analysis (*n* = 3).

Samples were bacteriologically examined at 0, 2, 4, 6, 8, and 10th days of storage for *S. aureus* count according to FDA [30].

### 2.9. Assessment of the Acceptability of Chicken Fillet Fortified with the Lyophilized HO Algal Extract

A total of 10 experienced panelists applied the evaluation at the Food Technology Department, City of Scientific Research and Technological Applications, New Borg El Arab, Egypt. Sensory evaluation was applied on a grilled un-inoculated chicken fillet fortified with lyophilized HO algal extract to evaluate its acceptability as a food additive. The first three groups of experiments [(CHF), (CHF/HO 4%), and (CHF/HO 6%)] were evaluated after grilling for sensory attributes.

The samples were maintained at room temperature 25 °C/10 min before evaluation. Panelists evaluated the chicken fillet for the degree of acceptability depending on the following criteria: color, odor, taste, texture, and overall acceptance (10 points/each item), with a scale ranging from 1 to 10, where 10 is more accepted as described by Hamad et al. [31]. In addition, the average sensory attribute data with its standard deviations were evaluated.

### 2.10. Statistical Analysis

All calculations were implemented based on SPSS, version 23 (IBM SPSS Statistics for Windows, IBM Corp., Armonk, NY, USA). The means ± standard error (SE) was used for the data analyses. One-way analysis of variance (ANOVA) using the Duncan test was used, where the probability was considered statistically significant when *p* < 0.01 or *p* < 0.05.

## 3. Results and Discussion

### 3.1. Prevalence of S. aureus in Chicken Fillet

One of the main demands that will be increased by 2050 is protein. Chicken meat constitutes a major protein part depending on the diet [32,33]. Unfortunately, contamination of chicken meat with food poisoning bacteria occurs during any step of processing [34]. *S. aureus* is a common food poisoning hazard in chicken meat that secretes a heat resistance toxin that affects human health.

In the current study, chicken meat samples were evaluated bacteriologically for the presence of *S. aureus*. Results in Table S1 revealed the occurrence of *S. aureus* in breast and thigh chicken meat samples, which was 92% and 84%, respectively. There was no significant difference (*p* > 0.05) between the prevalence of *S. aureus* in the breast and thigh of the chicken. These results were higher than those by Momtaz et al. [35], who isolated *S. aureus* from 22.77% of fresh raw chicken meats. Qian et al. [5] confirm *S. aureus* contamination of chicken meat in all processing plant steps. Meat contamination with *S. aureus* negatively impacts human health and causes serious diseases [36] because it secretes enterotoxins in food. These toxins are thermo-stable and resistant to gastrointestinal proteases [37].

In this study, chicken meat was sampled as an equivalent criterion to the buying of chicken for consumers from suppliers with a low hygienic level for the tools utilized to cut the chicken, poor sanitation levels, and frequent direct contact between the meat and market visitors. The high prevalence of *S. aureus* in the detected chicken samples in the present research is due to contamination of the tested samples with the pathogenic microorganisms anywhere in the supply chain from farm to market. Similarly, contamination can also occur during contact with the facility’s equipment, such as belts, grinders, and saws, or by contact with chicken meat handlers like knives and hand contact. Furthermore, due to abundance of proteins, carbohydrates, fats, vitamins, in chicken and its high-water holding capacity (WHC) allow the formation of a suitable conditions and environment for *S. aureus* contamination and growth.

### 3.2. Antibacterial Activity of Lyophilized Seaweed Extracts

Producing food free from foodborne diseases based on natural antimicrobials has become a great interest in food safety sector. Marine algae consider novel food additives to produce natural and functional products that fulfill consumer demand because of their secondary bioactive metabolites as phenolic compounds [38]. Moreover, it has antimicrobial effects on different forms of bacteria [39].

**Figure 2:**
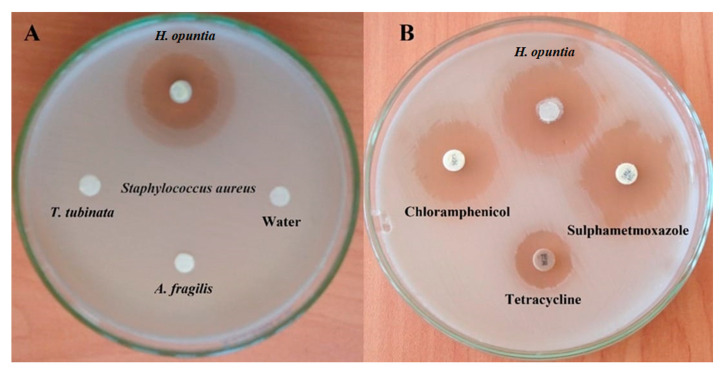
Antibacterial activity of lyophilized seaweed extracts (*Halimeda opuntia*, *Actinotrichia fragilis*, and *Turbinaria turbinata*) against *S. aureus* using agar disk diffusion assay vs. chloramphenicol, tetracycline, and sulfamethoxazole antibiotics. Inhibitions zones are measured in mm. (**A**) Antibacterial effect of three lyophilized seaweed extracts, (**B**) Antibacterial effect of antibiotics compared with *H. opunti*a.

This study evaluated the antibacterial effect of three lyophilized seaweed extracts on *S. aureus* using an agar disk diffusion assay. Results in Table 1, Figure 2A,B reveal a comparative study of in vitro antibacterial effects of lyophilized HO (green algae), lyophilized AF (red algae), and lyophilized TT (brown algae), as well as three types of antibiotics, were used as references against *S. aureus*. Furthermore, the lyophilized HO extract was the only algae that exhibited an anti-*S. aureus* effect with an inhibition zone of 43.16 ± 0.44 mm (Figure 2A) and even higher than those of chloramphenicol, tetracycline, and sulfamethoxazole antibiotics with a significant difference at *p* < 0.05 (Figure 2B). This result agrees with that of Ely et al. [40] and Manivannan et al. [41], who confirmed the algal extracts have in vitro antibacterial activity against Gram-positive and Gram-negative.

### 3.3. Minimum Inhibitory Concentrations (MICs) of Lyophilized HO Extract

**Figure 3:**
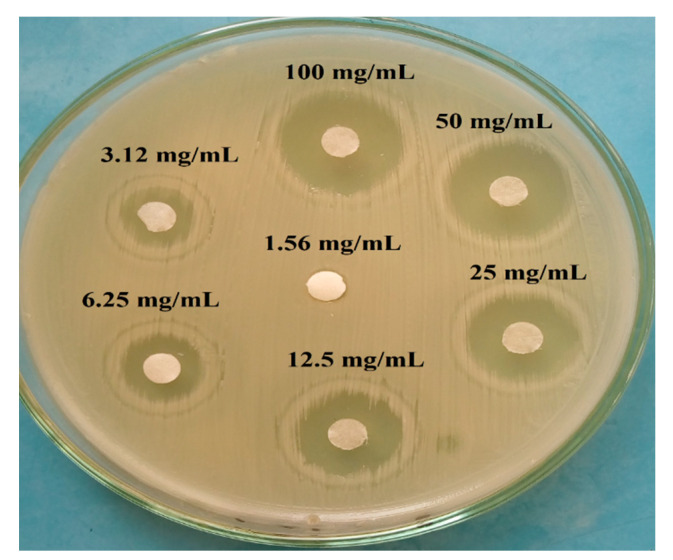
Determination of the minimum inhibitory concentration (MIC) of HO extract against *S. aureus* EMCC1351.

MIC of the lyophilized HO extract against *S. aureus* in vitro and the antibacterial effect of different concentrations was evaluated. Results in Table 1 and Figure 3 showed that lyophilized HO extracts at a minimum concentration of 3.12 mg/mL exhibited an anti-*S. aureus* with inhibition zone of 8.16 ± 0.73 mm. Furthermore, the anti-*S. aureus* activity increased relatively by the gradually increment of the extract concentration.

### 3.4. DPPH Radical Scavenging Capacity

The DPPH assay is an accurate, reliable, and cost-effective way to assess antioxidant radical scavenging activity. The antioxidant capacity of the lyophilized HO extract was evaluated in Table 2 depending on the DPPH radical scavenging capacity. Results compared with ascorbic acid as a standard antioxidant. It was found that the IC_50_ of ascorbic acid was 26.36 µg/mL while that of the lyophilized HO extract was 55.3 µg/mL. The highest DPPH scavenging activity of the lyophilized HO extract was 95.34% at a concentration of 100 µg/mL. These results were higher than those by Nazarudin et al. [42], who found that the HO highest DPPH reduction is 63.61% at 1000 mg/mL concentration. This seaweed radical scavenging ability enhances its antioxidant effect on carcinogenesis [43].

### 3.5. TPC and TFC of Lyophilized HO Extract

From the current findings, it was found that TPC was 20.36 ± 0.092 mg/mL, while TFC was 16.59 ± 0.029 mg/mL. These results were lower than those of Nazarudin et al. [40], who found that TPC and TFC of HO extracts are 55.04 ± 0.98 mg/g and 40.02 ± 0.02 mg/g, respectively. Many factors affect the variation in phenolic content: location, ecological classification, season, temperature, pH, light incidence, water salinity, and water nutrient composition [44]. The higher antioxidant potential (Table 2) is mainly due to the high level of TPC and TFC.

### 3.6. Phenolic Profile of Lyophilized HO Algal Extract by HPLC

**Figure 4:**
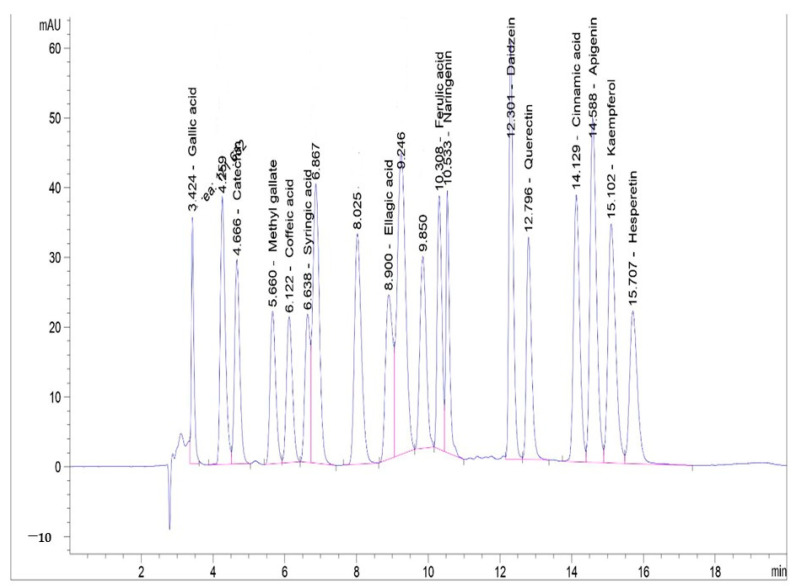
HPLC chromatogram of phenolic compounds profile of lyophilized HO algal extract. (1) Gallic acid, (3) Catechin, (4) Methyl gallate, (5) Caffeic acid, (6) Syringic acid, (9) Ellagic acid, (12) Ferulic acid, (13) Naringenin, (14) Daidzein, (15) Quercetin, (16) Cinnamic acid, (17) Apigenin, (18) Kaempferol, and (19) Hesperetin.

HPLC evaluates the phenolic profile content in marine microalgae [45]. Therefore, a detailed profile of the phenolic content of the lyophilized HO extract was illustrated by HPLC in Table S2 and Figure 4. It was found that lyophilized HO extract exhibited many phenolic bioactive compounds that could be explain its antioxidant and antibacterial activity. The highest bioactive compound was daidzein with 10.84 ± 0.005 µg/mL, followed by gallic acid with 4.06 ± 0.006 µg/mL. Indeed, daidzein has antioxidant and anticancer properties [46]. Gallic acid is used in the food industry as an antioxidant and preservative owing to its strong radical scavenging and antioxidant activities [47]. Moreover, it is a potent antimicrobial, gastroprotective, anticancer, antioxidant, promotes many health benefits to humans, and protects both the heart and kidney [48].

The determined phenolics could be attributed to the lyophilized HO extract’s antimicrobial and antioxidant roles proved in this study. Kurhekar, [49] refers to the antimicrobial effect of marine algae for its content of various bioactive compounds, e.g., ascorbic acid, phenolic acids, lutein, α -tocopherol, α -carotene, β-carotene, and flavonoids.

### 3.7. Safety and Cytotoxicity Assay of Lyophilized HO Algal Extract

The PBMCs cytotoxicity approach, utilizes cells isolated from multiple individuals, provides a high throughput evaluation of the cytotoxicity in vitro of candidate drugs. Additionally, PBMCs assay provide a primary reflection into how immune cell from different donors respond to the candidate compounds in development. Indeed, the safety of any new antimicrobials added to food is of great concern [42,50,51,52], therefore, the safety and/or cytotoxicity of the lyophilized HO extract were assessed. The cytotoxic effect of the lyophilized HO extract on the viability of PBMCs (Table S3) revealed that the cytotoxicity of PBMCs showed a positive correlation with the lyophilized HO extract concentration. The concentrations of lyophilized HO extract ranged from a maximum of 250 µg/mL to a minimum of 7.8 µg/mL, which showed inhibition of the viability of PBMCs with 69.27%, respectively. Moreover, the amount of lyophilized HO extract required to cause 50% inhibition of PBMC cells (IC_50_) was 33.7 µg/mL. A higher IC_50_ permits the use of lyophilized HO extract as a safe food additive in meat products.

### 3.8. Chicken Fillets Challenge Study

Regarding the rising chicken meat consumption global concern [53], the safety of chicken meat is considered a common consumer demand. In a challenge study, lyophilized HO algal extract was applied in chicken fillets experimentally inoculated with *S. aureus* to evaluate its antibacterial effect. Results in Table 3 revealed that lyophilized HO algal extract showed an anti-*S. aureus* effect in chicken fillets stored at 4 °C. CHF/ST/HO 4% and 6% caused a complete reduction of *S. aureus* to count on the 6th and 4th days of storage, while the count of *S. aureus* in the CHF/ST group gradually increased. Furthermore, there was a significant difference (*p* < 0.05) between treated and untreated groups with lyophilized HO across the entire storage period.

Chicken fillet without any treatments (CHF); chicken fillet treated with lyophilized HO algal extract 4% (CHF/HO 4%); chicken fillet treated with lyophilized HO algal extract 6% (CHF/HO 6%); chicken fillet experimentally inoculated with 10^7^ CFU/mL *S. aureus* (CHF/ST); chicken fillet experimentally inoculated with *S. aureus* and treated with lyophilized HO algal extract 4% (CHF/ST/HO 4%); and chicken fillet experimentally inoculated with *S. aureus* and treated with lyophilized HO algal extract 6% (CHF/ST/HO 6%).

The challenge study results go with the previous evaluation of the antioxidant and in vitro anti-*S. aureus* effect of lyophilized HO algal extract. The anti-*S. aureus* effects of lyophilized HO algal extract in chicken fillet stored at 4 °C may be attributed to the fact that marine algae contain phenolic compounds that compete against invading bacteria [54,55], it considers a broad-spectrum antiviral and antibacterial [56] those from the Arabian Gulf and the Saudi Arabia Red Sea inhibit methicillin-resistant *S. aureus* [45]. HO was not evaluated before against *S. aureus*. However, it activates the antibacterial effect of zinc oxide nanoparticles on pathogenic *Vibrio harveyi* [57]. Moreover, the lyophilized algal extract contains higher bioactive compounds and hence more antimicrobial activity [58]. Thus, it is considered a sustainable food for humans.

### 3.9. Acceptability of Chicken Fillet Fortified with Lyophilized HO Algal Extract

The sensory attributes of grilled un-inoculated chicken fillet fortified with lyophilized HO algal extract were evaluated. Results in Table 4 revealed that CHF/HO 4% and CHF/HO 6% enhanced the color, odor, taste, texture, and overall acceptability of grilled chicken fillet. Moreover, there was a significant difference (*p* < 0.05) between all treatments. Further, HO algae showed acceptability to consumers. The recent studies confirmed that, HO algae had pleasant organoleptic attributes, and even a protein alternative in meat products [59,60].

## 4. Conclusions

The current finding revealed that, the occurrence of *S. aureus* in tested breast and thigh chicken meat samples was recorded at 92% and 84%, respectively. There was no significant difference (*p* > 0.05) between the prevalence of *S. aureus* in the breast and thigh of the chicken. The high occurrence of *S. aureus* in the chicken samples detected in the present research is due to the contamination occurred by the pathogenic microorganisms anywhere in the supply chain, from farm to market.

This study evaluated the antibacterial effect of three lyophilized seaweed extracts on *S. aureus*. Amongst these three seaweeds, results demonstrated that HO extract was the only algae that exhibited an antibacterial activity against *S. aureus* due to the high content of total phenolic compounds, total flavonoids compounds, as well as several phenolic compounds illustrated by HPLC profile. In addition, HO extract exhibited antioxidant effect owing to the various bioactive molecules, which have the ability to scavenge free radicals (DPPH). A higher IC_50_ of HO extracts permits their use as a safe food additive in meat products. In a challenging study, the lyophilized HO extract displayed an anti-*S. aureus* effect in chicken fillets stored at 4 °C. HO extract also enhanced the sensory attributes of grilled un-inoculated chicken fillet. In sum, lyophilized HO extracts are promising food supplements in the chicken meat industry with eminent antibacterial and antioxidant properties.

## Figures and Tables

**Table 1:** Antibacterial activity and minimum inhibitory concentrations (MICs) of lyophilized seaweeds’ extracts against *S. aureus* using agar disk diffusion assay.

Extract/Material	Concentration/Volume	Inhibition Zone (mm) Against *S. aureus*
lyophilized HO extract (green algae)	100 mg/mL	43.16 ± 0.44 ^a^
lyophilized TT extract (brown algae)	100 mg/mL	NZ
lyophilized AF extract (red algae)	100 mg/mL	NZ
Water	20 µL	NZ
Tetracycline	30 mg/mL	15.26 ± 0.34 ^d^
Chloramphenicol	30 mg/mL	28.17 ± 0.42 ^b^
Sulphametmoxazole	100 mg/mL	23.33 ± 0.60 ^c^
Minimum inhibitory concentrations (MICs)		
Strain	lyophilized HO extract against *S. aureus* (mg/mL)	
*S. aureus*	Conc. (mg/mL)	Inhibition zone (mm)
	100	42.0 ± 0.28
	50	31.17 ± 0.43
	25	20.33 ± 0.72
	12.5	16.17 ± 0.44
	6.25	13.0 ± 0.26
	3.12	10.23 ± 0.57
	1.56	ND

NZ: No Zone; MIC: Minimum Inhibition Concentration; ND: Not detected; HO: *Halimeda opuntia*, AF: *Actinotrichia fragilis*; TT: *Turbinaria turbinata*. ^a,b,c,d^ Data in the same column followed by different superscript letters differ significantly (*p* < 0.05).

**Table 2:** DPPH radical scavenging capacity of lyophilized HO extract comparing with ascorbic acid as standard.

Conc. (µg/mL)	Ascorbic Acid		Lyophilized HO Extract (Green Algae)	
	Inhibition (%)	IC_50_ (µg/mL)	Inhibition (%)	IC_50_ (µg/mL)
10	5.12 ± 0.005 ^b^	26.36	9.62 ± 0.006 ^a^	55.36
20	35.19 ± 0.006 ^a^		18.51 ± 0.008 ^b^	
30	56.89 ± 0.007 ^a^		27.64 ± 0.007 ^b^	
40	80.03 ± 0.035 ^a^		35.53 ± 0.003 ^b^	
50	89.61 ± 0.003 ^a^		43.75 ± 0.006 ^b^	
60	94.72 ± 0.004 ^a^		54.19 ± 0.005 ^b^	
70	97.20 ± 0.005 ^a^		67.51 ± 0.004 ^b^	
80	98.68 ± 0.003 ^a^		80.23 ± 0.007 ^b^	
90	99.34 ± 0.004 ^a^		89.63 ± 0.006 ^b^	
100	99.67 ± 0.002 ^a^		95.34 ± 0.011 ^b^	

^a,b^ Data in the same row between different antioxidant activity (%) followed by different superscript letters differ significantly (*p* < 0.01).

**Table 3:** Antibacterial effect of different concentration from lyophilized HO algal extract against *S. aureus* experimentally inoculated in chicken fillet stored at 4 °C (mean ± SE).

Storage (Days)	CHF	CHF/HO 4%	CHF/HO 6%	CHF/ST	CHF/ST/HO 4%	CHF/ST/HO 6%
0	0.00	0.00	0.00	7.04 ± 0.022 ^Aa^	7.04 ± 0.022 ^Aa^	7.04 ± 0.022 ^Aa^
2nd	0.00	0.00	0.00	7.18 ± 0.016 ^Ba^	6.71 ± 0.008 ^Bab^	5.78 ± 0.004 ^Bb^
4th	0.00	0.00	0.00	7.32 ± 0.020 ^Ca^	4.38 ± 0.00 ^Cb^	2.61 ± 0.008 ^Cc^
6th	0.00	0.00	0.00	7.40 ± 0.021 ^Da^	3.49 ± 0.014 ^Db^	0.00 ^Dc^
8th	0.00	0.00	0.00	7.50 ± 0.007 ^Ea^	0.00 ^Eb^	0.00 ^Db^
10th	0.00	0.00	0.00	7.72 ± 0.005 ^Fa^	0.00 ^Eb^	0.00 ^Db^

CHF: chicken fillet without any treatments, CHF/HO 4%: chicken fillet treated with HO algal extract 4%, CHF/HO 6%: chicken fillet treated with lyophilized HO algal extract 6%, CHF/ST: chicken fillet experimentally inoculated with 10^7^ CFU/mL *S. aureus* CHF/ST/HO 4%: chicken fillet experimentally inoculated with *S. aureus* and treated with lyophilized HO algal extract 4%, and CHF/ST/HO 6%: chicken fillet experimentally inoculated with *S. aureus* and treated with lyophilized HO algal extract 6%. *S. aureus* counts are in (Log10 CFU/g). ^A,B,C,D,E,F^ Data in the same column between same treatment at different storage periods followed by different superscript letters differ significantly (*p* < 0.05). ^a,b,c^ Data in the same row between different treatments at same storage periods followed by different superscript letters differ significantly (*p* < 0.05).

**Table 4:** Acceptability of grilled un-inoculated chicken fillet fortified with lyophilized HO algal extract depending on sensory attributes.

Samples	Color	Odor	Taste	Texture	Overall Acceptance
CHF	8.00 ± 0.13 ^B^	7.85 ± 0.29 ^C^	8.20 ± 0.25 ^B^	8.00 ± 0.27 ^B^	8.30 ± 0.15 ^B^
CHF/HO 4%	8.50 ± 0.15 ^A^	8.05 ± 0.16 ^B^	8.65 ± 0.15 ^A^	8.45 ± 0.17 ^A^	8.44 ± 0.14 ^A^
CHF/HO 6%	8.35 ± 0.19 ^A^	8.50 ± 0.13 ^A^	8.70 ± 0.11 ^A^	8.50 ± 0.14 ^A^	8.65 ± 0.11 ^A^

CHF: chicken fillet without any treatments; CHF/HO 4%: chicken fillet treated with lyophilized HO algal extract 4%; CHF/HO 6%: chicken fillet treated with lyophilized HO algal extract 6%. ^A,B,C^ Data in the same column between different treatment followed by different superscript letters differ significantly (*p* < 0.05).

## Data Availability

The data presented in this study are available on request from the corresponding author.

## Supplementary Materials

The following supporting information can be downloaded at: https://www.mdpi.com/article/10.3390/foods12010020/s1, Table S1. Prevalence of S. aureus in chicken fillet collected from different local markets (n = 100); Table S2. HPLC evaluation for phenolic profile of lyophilized HO algal extract; Table S3. Evaluation of safety and cytotoxicity assay to lyophilized HO algal extract on the viability of PBMCs cells.
