# Supplementary material for: Evaluation of the Prevalence of Staphylococcus aureus in Chicken Fillets and Its Bio-Control Using Different Seaweed Extracts
Source: Foods. 2022 Dec 21;12(1):20. doi: 10.3390/foods12010020 (PMC9818820; doi:10.3390/foods12010020)
Supplement: Supplementary file 1 [file foods-12-00020-s001.zip › foods-2083129-supplementary.pdf]

Table S1. Prevalence of *S. aureus* in chicken fillet collected from different local markets (*n*= 100).

Types of Chicken Fillets	Negative Samples		Positive Samples	
	**No.**	**%**	**No.**	**%**
Breast (n=50)	4	8	46	92
Thigh (n=50)	8	16	42	84
Total (n=100)	12	12	88	88
Note: Chi^2= 1.515 *p*-value >0.05				

Table S2. HPLC evaluation for phenolic profile of lyophilized HO algal extract.

Phenolic Compounds	RT	Concentration (μg/mL)
Gallic acid	3.424	4.06
Catechin	4.666	0.73
Methyl gallate	5.660	0.23
Caffeic acid	6.122	1.78
Syringic acid	6.638	2.35
Ellagic acid	8.900	2.91
Ferulic acid	10.308	0.53
Naringenin	10.533	0.92
Daidzein	12.301	10.84
Quercetin	12.796	0.96
Cinnamic acid	14.129	0.89
Apigenin	14.588	2.79
Kaempferol	15.102	1.53
Hesperetin	15.707	0.96

RT: retention time (min).

Table S3. Cytotoxicity evaluation of lyophilized HO algal extract on the viability of peripheral blood mononuclear cells (PBMCs).

**Concentration (μg/mL)**	**Inhibition %**	**Viability %**
250	69	31
125	69	31
62.5	64	36
31.2	30	70
15.6	29	71
7.8	27	73
IC_{50}	33.7 μg/mL	
